# *Cissus subtetragona* Planch. Ameliorates Inflammatory Responses in LPS-induced Macrophages, HCl/EtOH-induced Gastritis, and LPS-induced Lung Injury via Attenuation of Src and TAK1

**DOI:** 10.3390/molecules26196073

**Published:** 2021-10-08

**Authors:** Laily Rahmawati, Nur Aziz, Jieun Oh, Yo Han Hong, Byoung Young Woo, Yong Deog Hong, Philaxay Manilack, Phetlasy Souladeth, Ji Hwa Jung, Woo Shin Lee, Mi Jeong Jeon, Taewoo Kim, Mohammad Amjad Hossain, Jinwhoa Yum, Jong-Hoon Kim, Jae Youl Cho

**Affiliations:** 1 Department of Integrative Biotechnology, Sungkyunkwan University, Suwon 16419, Korea; lyrahma0106@g.skku.edu (L.R.); nuraziz@skku.edu (N.A.); martia96@gmail.com (J.O.); ghddygks13@naver.com (Y.H.H.); 2 AmorePacific R&D Center, Yongin 17074, Korea; quddud@amorepacific.com (B.Y.W.); hydhong@amorepacific.com (Y.D.H.); 3 Department of Forestry, Ministry of Agriculture and Forestry, Vientiane P.O. Box 811, Laos; pmanilack@gmail.com; 4 Department of Forest Management, Faculty of Forest Science, National University of Laos, Vientiane P.O. Box 7322, Laos; p.souladeth@nuol.edu.la; 5 Division of Zoology, Honam National Institute of Biological Resources, Mokpo 58762, Korea; biyam8712@naver.com; 6 Department of Forest Sciences, College of Agriculture and Life Science, Seoul National University, Seoul 08826, Korea; krane@snu.ac.kr; 7 Animal Resources Division, National Institute of Biological Resources, Incheon 22689, Korea; jeonmj@korea.kr (M.J.J.); pulmuchi@korea.kr (T.K.); lestes93@korea.kr (J.Y.); 8 Department of Veterinary Physiology, College of Medicine, Chonbuk National University, Iksan 54596, Korea; mamjadh2@gmail.com

**Keywords:** *Cissus subtetragona* Planch, inflammation, Src, TAK1, gastritis, acute lung injury

## Abstract

Several *Cissus* species have been used and reported to possess medicinal benefits. However, the anti-inflammatory mechanisms of *Cissus subtetragona* have not been described. In this study, we examined the potential anti-inflammatory effects of *C. subtetragona* ethanol extract (Cs-EE) in vitro and in vivo, and investigated its molecular mechanism as well as its flavonoid content. Lipopolysaccharide (LPS)-induced macrophage-like RAW264.7 cells and primary macrophages as well as LPS-induced acute lung injury (ALI) and HCl/EtOH-induced acute gastritis mouse models were utilized. Luciferase assays, immunoblotting analyses, overexpression strategies, and cellular thermal shift assay (CETSA) were performed to identify the molecular mechanisms and targets of Cs-EE. Cs-EE concentration-dependently reduced the secretion of NO and PGE_2_, inhibited the expression of inflammation-related cytokines in LPS-induced RAW264.7 cells, and decreased NF-κB- and AP-1-luciferase activity. Subsequently, we determined that Cs-EE decreased the phosphorylation events of NF-κB and AP-1 pathways. Cs-EE treatment also significantly ameliorated the inflammatory symptoms of HCl/EtOH-induced acute gastritis and LPS-induced ALI mouse models. Overexpression of HA-Src and HA-TAK1 along with CETSA experiments validated that inhibited inflammatory responses are the outcome of attenuation of Src and TAK1 activation. Taken together, these findings suggest that Cs-EE could be utilized as an anti-inflammatory remedy especially targeting against gastritis and acute lung injury by attenuating the activities of Src and TAK1.

## 1. Introduction

The essential role of inflammation as part of the innate immune response has been widely reported. Microbial infection, as well as tissue stress or damage, can induce an inflammatory reaction via recognition of either damage-associated molecular patterns (DAMPs) or pathogen-associated molecular patterns (PAMPs). Macrophages, one type of immune cells, play a critical role in pathogen recognition since they highly express Toll-like receptors (TLRs) [1]. TLRs are one of the most substantial and represented pattern recognition receptors (PRRs). Engagement of a particular PAMP into these receptors ultimately induces intracellular signaling cascades that together orchestrate the early host response to infection, followed by subsequent activation and shaping of adaptive immunity [1,2]. At the molecular level, engagement of PAMPs such as lipopolysaccharides (LPS) derived from Gram-negative bacteria into TLR-4 will induce recruitment of adaptor proteins into the cytoplasm. This activates molecular cascades that are immediately transduced via signal transduction pathways including a protein tyrosine kinase, Src, and a MAPK kinase kinase (MAPKKK), TAK1, to mediate nuclear translocation and activate transcription factors such as nuclear factor (NF)-κB and activator protein (AP)-1 [3,4,5]. Activation of these transcription factors induces the expression of mRNA of inflammatory genes such as inducible nitric oxide synthase (iNOS), cyclooxygenase-2 (COX-2), interleukin (IL)-6, IL-1β, and tumor necrosis factor-alpha (TNF-α), subsequently releasing the production of the inflammatory mediators, prostaglandin E_2_ (PGE_2_) and nitric oxide (NO), and various cytokines [1,4,6]. Thus, understanding these signaling events will potentially provide the opportunity to prevent and remedy various inflammatory diseases. An inflammatory response has been recognized to be an essential process for host defense mechanisms. On the other hand, excessive inflammation or chronic inflammation has been reported to be associated with chronic inflammatory diseases such as autoimmune diseases or cancer development and progression [7,8,9]. Therefore, regulating inflammatory responses through anti-inflammatory agents is considered as a method for preventing the development of various diseases.

Some natural products have been extensively studied and reported to have anti-inflammatory activity. Over the last several years, our research group has studied traditional medicinal plants to develop safer and more efficient anti-inflammatory therapies that target specific intracellular signaling events [6,10,11]. *Cissus subtetragona* Planch., distributed in Guangdong, Guangxi, Hainan, Yunnan [Laos, Vietnam] and locally known in China as “si leng bai fen teng”, is a liana, woody plant species that belongs to the genus *Cissus* (family Vitaceae) with a least known pharmacological activity but a lack of scientific study reports. This plant is medicinally used for sore throat in Southwest China [12], and is also widely used in Luang Prabang, Northern Laos [13]. Hence, we sought to see the potential of this plant in a wide context of treating inflammatory diseases. Additionally, ethnopharmacological evidence suggests that at least a dozen plants from the genus *Cissus,* such as *C. quadrangularis*, *C. assamica*, and *C. verticillata,* have been used for treating diseases such as obesity, diabetes, fracture, rheumatic arthritis, gastrointestinal problems, urinary problems, and various inflammatory diseases in traditional medicines in different parts of the world [14,15]. In this study, for the first time, we examined the potential anti-inflammatory activity of *C. subtetragona* ethanol extract (Cs-EE) using in vitro (LPS-activated macrophages) and in vivo conditions (LPS-induced acute lung injury (ALI) and HCl/EtOH-induced gastritis models), commonly mediated by TLR activation pathway via PAMPs (activated macrophages and LPS-induced ALI) and DAMPs (HCl/EtOH-induced mucosal injury of the stomach) [1]. The molecular targets and the chemical constituents of Cs-EE were also investigated. 

## 2. Results

### 2.1. Cs-EE Affects NO and PGE_2_ Production without Showing Any Cytotoxicity

**Figure 1:**
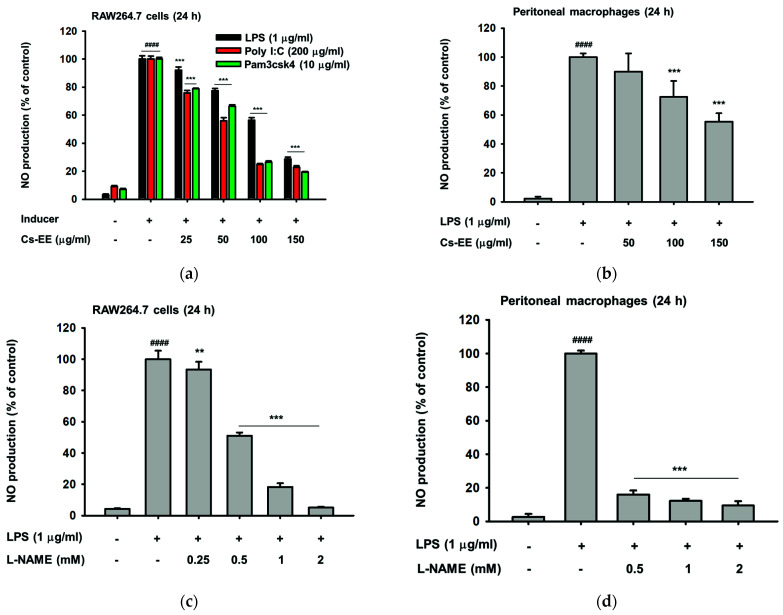

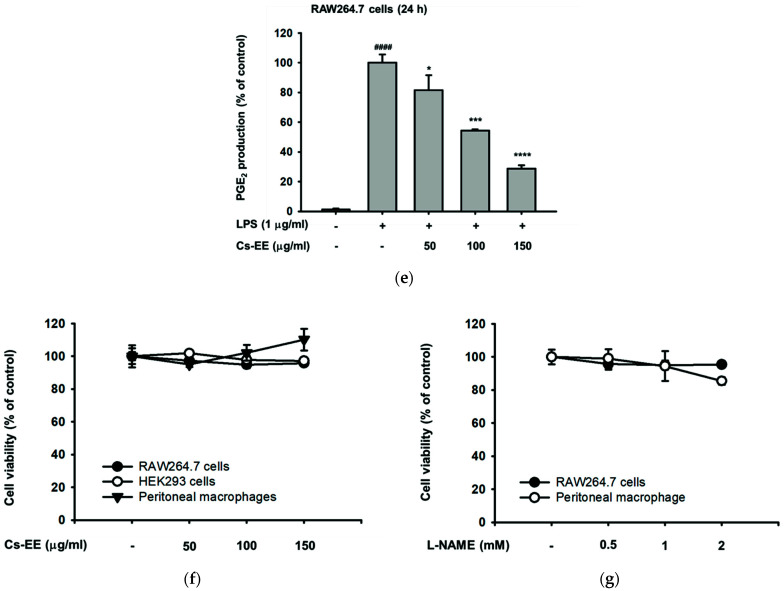
Effect of Cs-EE on the production of inflammatory mediators, and cell viability profile. (**a**–**d**) Supernatant NO levels in RAW264.7 cell cultures and peritoneal macrophages pre-treated with indicated concentrations of Cs-EE (**a**,**b**) or L-NAME (**c**,**d**) in the presence or absence of LPS (1 μg/mL), poly (I:C) (200 μg/mL), or pam3csk4 (10 μg/mL) were determined using the Griess assay. (**e**) Supernatant PGE_2_ levels in LPS-treated RAW264.7 cells were evaluated by EIA. (**f**,**g**) Cell viability of RAW264.7 cells, HEK293 cells, and peritoneal macrophages treated with Cs-EE (**f**) or L-NAME (**g**) for 24 h were analyzed using the MTT assay. Results (**a**–**g**) are expressed as mean ± SD. ^####^ *p* < 0.0001 compared to normal group (no treatment) by the student’s *t*-test, and * *p* < 0.05, ** *p* < 0.01, *** *p* < 0.01 and **** *p* < 0.001 compared to control group (inducer alone) by one-way ANOVA.

To screen whether Cs-EE has anti-inflammatory potential, we initially measured the secretion levels of inflammatory mediators such as PGE_2_ and NO, chemical markers of inflammatory responses, with different concentrations of Cs-EE. As shown in Figure 1a,b, the generation of NO was significantly (*p* < 0.0001) enhanced up to 30 fold changes by inflammation inducers such as LPS (ligand of TLR4) derived from Gram-negative bacteria, pam3csk4 (ligand of TLR1/2) derived from Gram-positive-bacteria, or poly(I:C) (ligand of TLR3) derived from a virus. Conversely, pre-treatment with Cs-EE remarkably and concentration-dependently reduced NO production up to 75% after treatment with 150 μg/mL of Cs-EE in macrophage-like RAW264.7 cells (Figure 1a) and primary peritoneal macrophages (Figure 1b). As a positive control, the inhibitory effects of the standard NO inhibitor, L-NAME, were also examined. As shown in Figure 1c,d, L-NAME significantly (*p* < 0.001) decreased NO production in a concentration-dependent manner in LPS-treated RAW264.7 cells (Figure 1c) and peritoneal macrophages (Figure 1d). Additionally, Cs-EE also effectively and concentration-dependently (*p* < 0.05 at 50 μg/mL and *p* < 0.0001 at 150 μg/mL) reduced LPS-triggered PGE_2_ in RAW264.7 cells (Figure 1e). Importantly, using a conventional MTT assay, it was found that treatment with Cs-EE (Figure 1f) or with L-NAME (Figure 1g) did not induce cytotoxicity (there was no interference of cell viability up to 5%) in RAW264.7 cells, HEK293 cells, or peritoneal macrophages, indicating that inhibition of NO and PGE_2_ by Cs-EE was not due to induction of cell death.

### 2.2. The Phytochemical Constituents of Cs-EE

**Figure 2:**
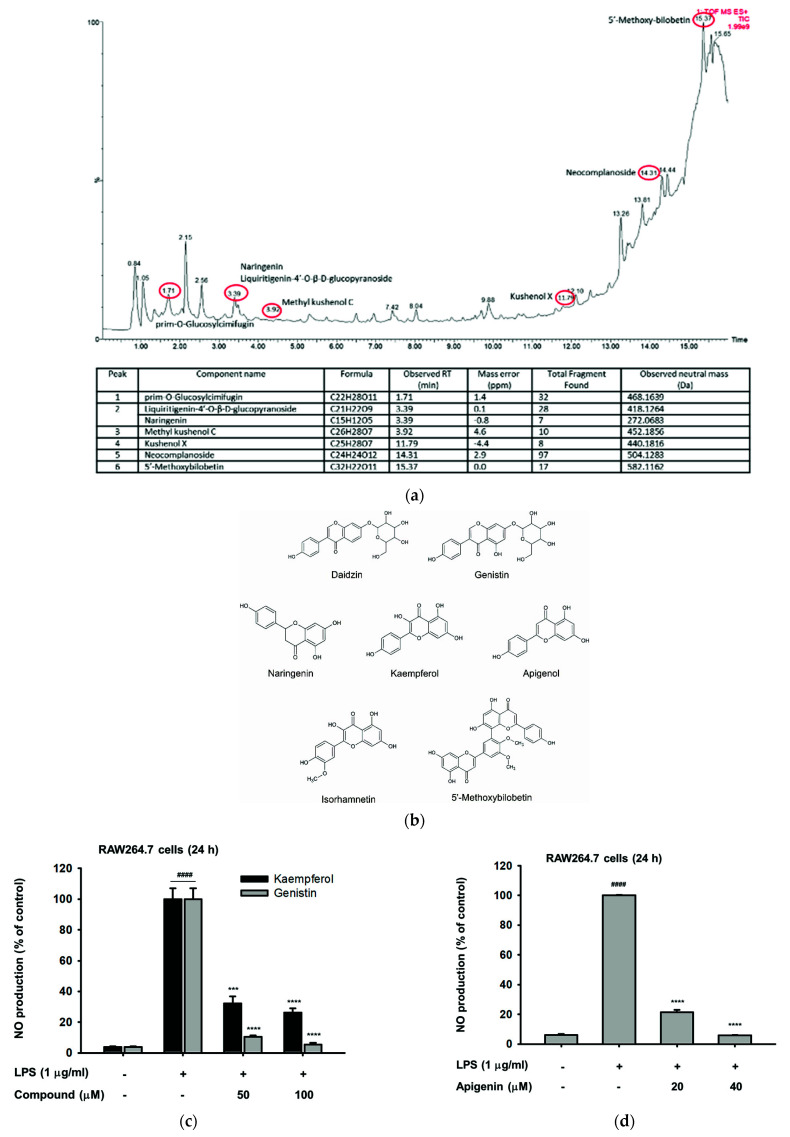
The phytochemical constituents of Cs-EE. (**a**) LC/MS-MS chromatogram of Cs-EE. (**b**) Chemical structure of the well-known flavonoids that was identified in Cs-EE. (**c**,**d**) Supernatant NO levels in RAW264.7 cell cultures pre-treated with indicated concentrations of ingredient compounds (kaempferol, genistin, and apigenin) in the presence or absence of LPS (1 μg/mL), were determined using the Griess assay. Results (**b**,**c**) are expressed as mean ± SD. ^####^ *p* < 0.0001 compared to normal group (no treatment), *** *p* < 0.001 and **** *p* < 0.0001 compared to control group (LPS alone). A *p*-value was analyzed using the student’s *t*-test.

Furthermore, we performed LC/MS-MS analysis to identify the phytochemical composition, especially pertaining to flavonoids, of Cs-EE. Peaks were observed at the retention time of 1.71 (prim-*O*-Glucosylcimifugin), 3.39 (naringenin and Liquiritigenin-4′-*O*-β-d-glucopyranoside), 3.92 (methyl kushenol C), 11.7 (kushenol X), 14.31 (neocomplanoside), and 15.37 (5′-Methoxybilobetin) as indicated in Figure 2a. More than 70 components from different flavonoid classes such as flavonols, flavones, isoflavones, biflavones, flavanones, and associated glycosides have been characterized and identified in Cs-EE (Supplementary Table S1), including kaempferol, isorhamnetin, naringenin, genistin, daidzin, and apigenol (apigenin) (Figure 2b). Kaempferol, genistin, and apigenin, which are known to possess anti-inflammatory activity, were also shown to strongly decrease the production level of NO in LPS-stimulated RAW264.7 cells (Figure 2c,d) without showing marked cytotoxicity (data not shown).

### 2.3. Effects of Cs-EE on the Expression of Inflammatory Genes and Transcriptional Activation of NF-κB and AP-1

**Figure 3:**
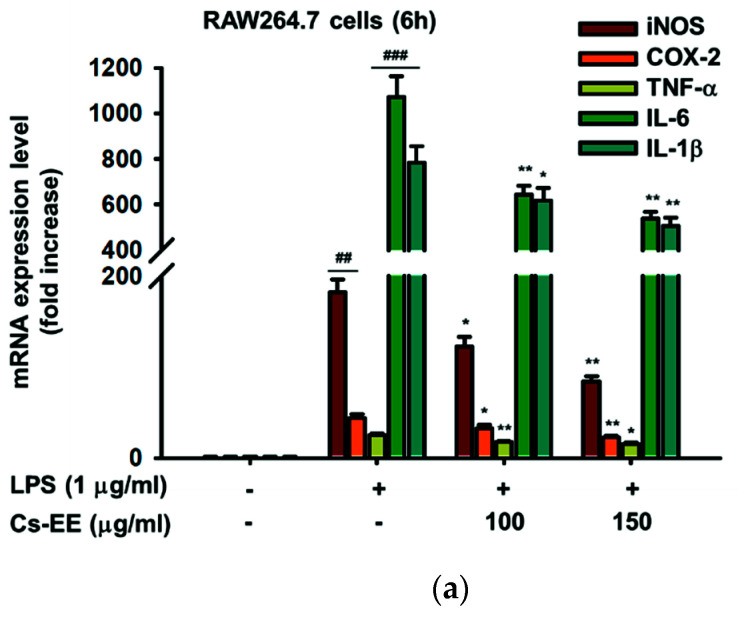

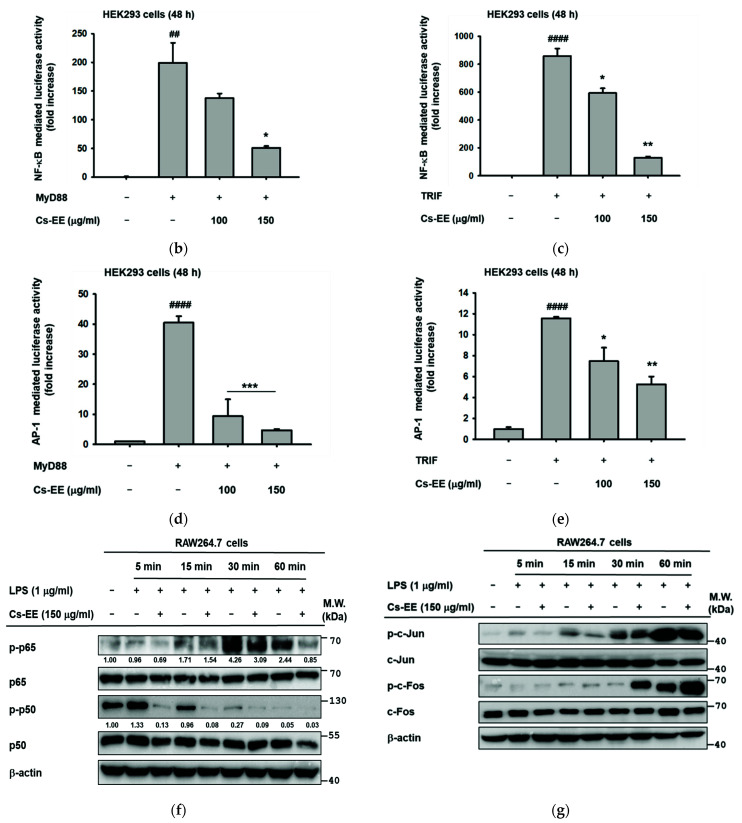
Effect of Cs-EE on the expression of inflammatory genes and transcriptional activation of NF-κB and AP-1. (**a**) TNF-α, IL-6, and IL-1β were analyzed by qPCR in LPS-treated RAW264.7 cells. (**b**–**e**) HEK293 cells were co-transfected with NF-κB- or AP-1-Luc, as well as β-gal (0.8 μg) together with MyD88 and TRIF for 48 h in the presence or absence of Cs-EE (100 and 150 μg/mL) before being measured using a luminometer. (**f**) The phospho- and total forms of NF-κB subunits, p50 and p65. The relative intensity values bellow the blot represent the quantification of band intensity which was measured by ImageJ and calculated as follows: (phospho-form of p65 or p50)/(p65 or p50). (**g**) The phospho- and total forms of AP-1 subunits, c-Jun and c-Fos, and β-actin from whole-cell lysates from LPS-treated RAW264.7 cells in the presence or absence of 150 μg/mL of Cs-EE were determined by immunoblot analysis. Results (**a**–**e**) are expressed as mean ± SD. ^##^ *p* < 0.01 and ^####^ *p* < 0.0001 compared to normal group by the student’s *t*-test, and * *p* < 0.05, ** *p* < 0.01 and *** *p* < 0.001 compared to control group (inducer alone) by one-way ANOVA.

Since we previously found that pre-treatment of LPS-treated RAW264.7 cells with Cs-EE can decrease inflammatory mediators, NO and PGE_2_, for the next step we also analyzed mRNA expression of iNOS, COX-2, and other pro-inflammatory genes such TNF-α, IL-6, and IL-1β. Assessment of mRNA levels was conducted using qRT-PCR. As expected, mRNA levels of inflammatory genes were upregulated by LPS. Additionally, in agreement with Figure 1, LPS-stimulated RAW264.7 cells pretreated with Cs-EE up to 150 μg/mL decreased mRNA levels of iNOS and COX-2 (*p* = 0.009 and *p* = 0.008, respectively) in a concentration-dependent manner (Figure 3a). Interestingly, other cytokine genes such as TNF-α, IL-6, and IL-1β also shown concentration-dependent decreases by pre-treatment with Cs-EE up to 150 μg/mL (*p* = 0.016, *p* = 0.001, and *p* = 0.009, respectively) (Figure 3a). Further, luciferase assays in HEK293 cells indicated that Cs-EE suppressed NF-κB and AP-1 luciferase activity induced by both MyD88 and TRIF conditions (Figure 3b–e). In addition, we also reevaluated the level of phosphorylation of p65 and p50, NF-κB subunits, as well as c-Jun and c-Fos, AP-1 subunits, from whole lysates in LPS-stimulated RAW264.7 cells at various time points. This experiment showed that Cs-EE reduced the level of p-p65 and p-p50 at all time points (Figure 3f), which indicated that Cs-EE could inhibit the activity of NF-κB. Additionally, Cs-EE decreased the level of p-c-Jun at 5, 15, and 60 min, but phosphorylation levels of c-Fos were not inhibited by Cs-EE (Figure 3g), which indicated that Cs-EE could inhibit the activity of AP-1 by inhibiting the dimerization of AP-1 via the reduction of activation of c-Jun.

### 2.4. Effects of Cs-EE on the Signaling Events Upstream of NF-κB Activation

**Figure 4:**
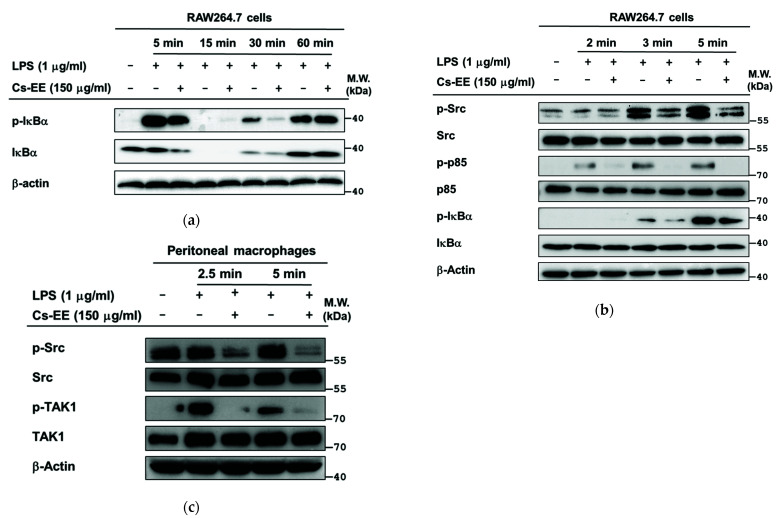

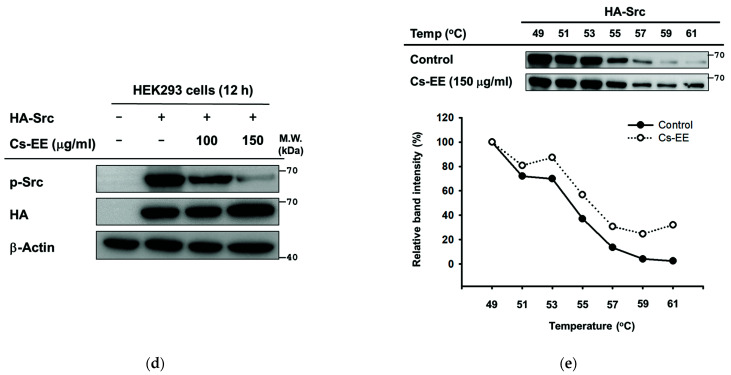
Effects of Cs-EE on the signaling events upstream of NF-κB activation. RAW264.7 cells (**a**,**b**) or peritoneal macrophages (**c**) were pre-treated with 150 μg/mL Cs-EE for 30 min, followed by with or without LPS induction at certain time points. The phosphorylated and total protein levels of IκBα, p85, Src, TAK1, and β-actin were evaluated by immunoblot analysis. (**d**) The phosphorylation of Src was evaluated in HEK293 cells following Src overexpression by transfection of HA-Src construct for 24 h and then treatment with Cs-EE (100 and 150 μg/mL). (**e**) CETSA was conducted in Src-overexpressing HEK293 cells treated with DMSO (control) or Cs-EE (150 μg/mL) at different heating temperatures. The interaction between Cs-EE and Src was analyzed by immunoblot analysis, and then quantification of band intensity was measured by ImageJ.

Next, in order to identify the molecular target of Cs-EE, we examined the upstream signaling molecules of NF-κB and AP-1 by immunoblot analysis. First, we checked the effects of Cs-EE on the signaling events upstream of NF-κB activation. p-IκBα was suppressed by Cs-EE at 5 and 30 min (Figure 4a) after LPS stimulation in RAW264.7 cells. Subsequently, we evaluated the upstream proteins of IκBα, p85, and Src kinase in brief time points. This showed that Cs-EE attenuated the phosphorylation level of Src (Tyr416) at early time points (3 and 5 min) together with a suppression of p-p85 and p-IκBα levels at 2, 3, and 5 min (Figure 4b). Using primary peritoneal macrophages, we also demonstrated that Cs-EE attenuated p-Src at early time points (2.5 and 5 min) (Figure 4c). These findings indicate that Src could be a putative target protein of Cs-EE in the NF-κB pathway. To confirm this hypothesis, Src-overexpressing HEK293 cells were evaluated. As expected, Cs-EE markedly reduced Src autophosphorylation in a concentration-dependent manner, as shown by immunoblot analysis (Figure 4d). Moreover, to evaluate the interaction of Cs-EE with Src, cellular thermal shift assay (CETSA) was performed at 7 different temperatures, ranging from 49–61 °C, in Src-overexpressing HEK293 cells. The Src protein was clearly observed in Cs-EE-treated groups compared to control (DMSO)-treated groups (Figure 4e). 

### 2.5. Effects of Cs-EE on the Signaling Events Upstream of AP-1 Activation

**Figure 5:**
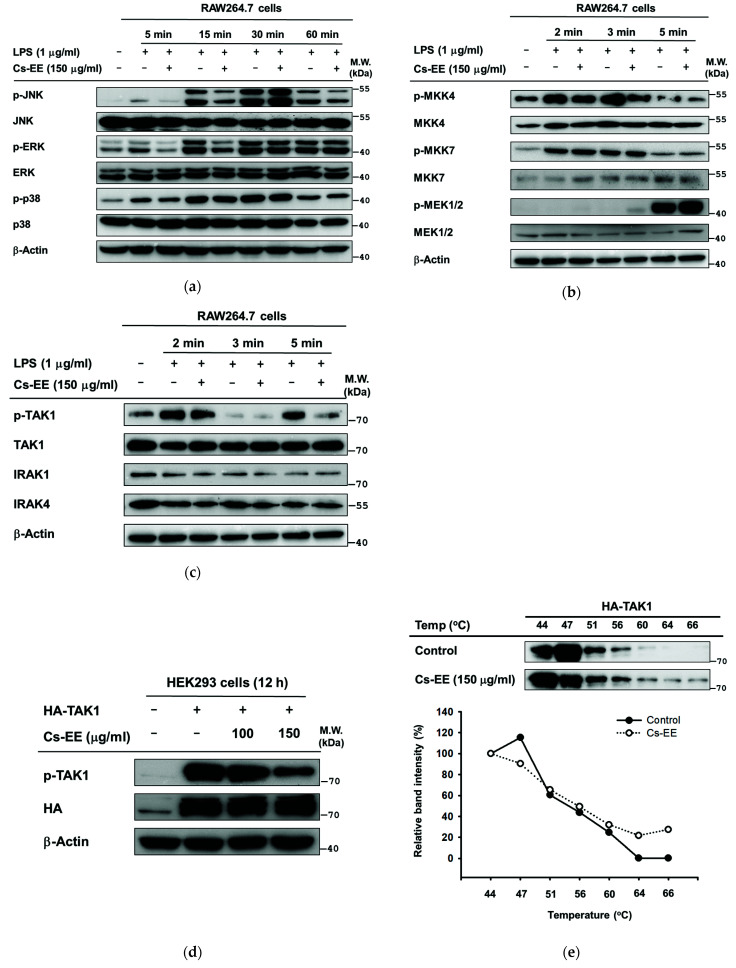
Effects of Cs-EE on the signaling events upstream of AP-1 activation. (**a**–**c**) RAW264.7 cells were pre-treated with 150 μg/mL Cs-EE for 30 min, followed by the presence or absence of LPS at certain time points. The phosphorylated and total protein levels of JNK, ERK, p38, MKK4/7, MEK1/2, TAK1, IRAK1/4, and β-actin were evaluated by immunoblot analysis. (**d**) The phosphorylation of TAK1 was evaluated in HEK293 cells following overexpression of TAK1 by transfection of HA-TAK1 construct for 24 h and then treatment with Cs-EE (100 and 150 μg/mL). (**e**) CETSA was conducted in TAK1-overexpressing HEK293 cells treated with Cs-EE (150 μg/mL) or control (DMSO) at different heating temperatures as indicated. The interaction between Cs-EE and TAK1 was analyzed by immunoblot analysis, and then quantification of band intensity was measured by ImageJ.

Additionally, we determined the target molecule of Cs-EE in AP-1 activation. MAPKs activation was enhanced by LPS-stimulated RAW264.7 cells at different time points. Among MAPKs, c-Jun N-terminal kinase (JNK) phosphorylation and extracellular signal-regulated kinase (ERK) were downregulated by at least the 5 min time point, but phosphorylation levels of p38 were not affected by Cs-EE (Figure 5a). Next, proteins upstream of JNK and ERK were evaluated at earlier time points. Only p-MKK4 was affected by CS-EE at 2, 3, and 5 min (Figure 5b). Consequently, we checked TAK1, one of the enzymes upstream of MAPKKs, together with IRAK1/4 at the same time points. Cs-EE regulated activated TAK1 (phosphorylated on ser412), but could not prevent the degradation of IRAK1/4 by LPS (Figure 5c). Using primary peritoneal macrophages, we also found that Cs-EE strongly attenuated p-TAK at the earlier times (2.5 and 5 min) (Figure 4c). Thus, we postulated that TAK1 could be a target protein of Cs-EE in the AP-1 pathway. To confirm this, TAK1-overexpressing HEK293 cells were examined. As expected, Cs-EE decreased TAK1 autophosphorylation in a concentration-dependent manner (Figure 5d). In addition, to understand the interaction of Cs-EE with TAK1, CETSA was also conducted at 7 different temperatures, ranging from 44‒66 °C, in TAK1-overexpressing HEK293 cells. The TAK1 protein was readily observable in Cs-EE-treated groups, meanwhile, the DMSO-treated control group exhibited different pattern (Figure 5e).

### 2.6. Effects of Cs-EE on HCl/EtOH-Induced Acute Gastritis Models 

**Figure 6:**
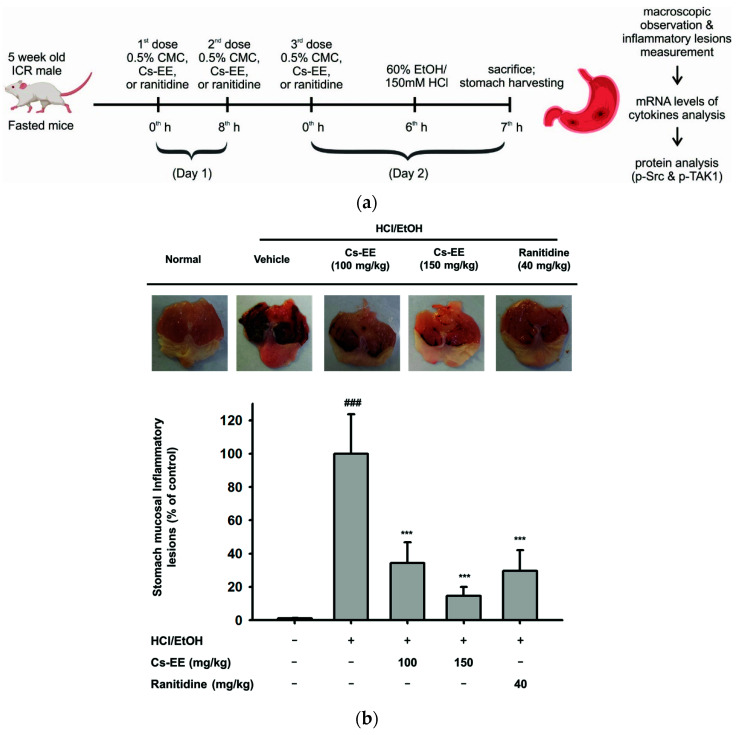

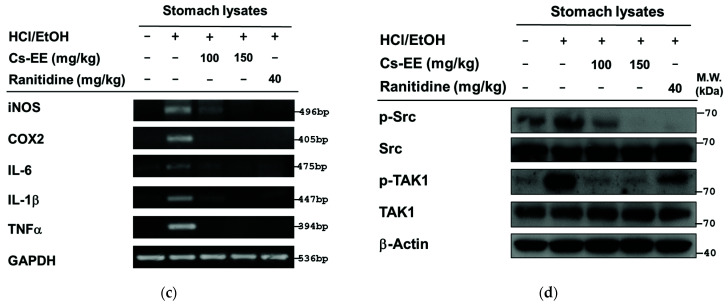
Effects of Cs-EE in the in vivo HCl/EtOH-induced acute gastritis models. (**a**) Schematic of HCl/EtOH-induced acute gastritis protocol. Fasted ICR mice were orally administrated with 100 μL different solutions based on different groups: normal group (0.5% CMC), HCl/EtOH group or as control group (0.5% CMC), Cs-EE groups (100 and 150 mg/kg Cs-EE) and ranitidine group (40 mg/kg ranitidine) three times in two days, and 150 mM HCl/60% EtOH was orally administrated (200 μL/mouse) 1 h before sacrifice. (**b**) The representative photograph of gastric inflammatory lesions, which then was quantified by ImageJ. Results (**b**) are expressed as mean ± SD (n = 5). ^###^ *p* < 0.001 compared to normal group, and *** *p* < 0.001 compared to control group (inducer alone). A *p*-value was analyzed using the student’s *t*-test. (**c**) The expression of inflammatory genes in stomach lysates was analyzed by semiquantitative RT-PCR. (**d**) The phospho- and total forms of Src, TAK1, and β-actin were evaluated by immunoblot analysis.

To examine the anti-inflammatory efficacy of Cs-EE in vivo, the HCl/EtOH-induced acute gastritis mouse model was induced using oral administration as illustrated in Figure 6a. Predictably, HCl/EtOH-induced gastritis showed prominent stomach mucosal inflammatory lesions. On the other hand, the 150 mg/kg Cs-EE groups significantly decreased (*p* < 0.001) and had the lowest stomach mucosal inflammatory lesions compared to ranitidine or 100 mg/kg Cs-EE groups (Figure 6b). In stomach samples, the level of inflammatory genes was decreased by Cs-EE (Figure 6c). We also examined phosphorylated Src and TAK1 in stomach lysates and found that p-Src and TAK1 were strongly inhibited by treatment with Cs-EE up to 150 mg/kg. Treatment with 40 mg/kg ranitidine also showed a reduction of these proteins (Figure 6d).

### 2.7. Effects of Cs-EE on LPS-Induced Acute Lung Injury Models

**Figure 7:**
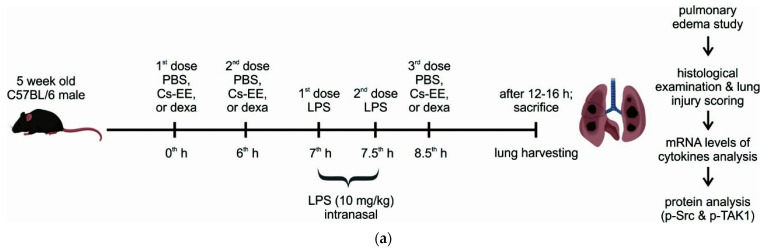

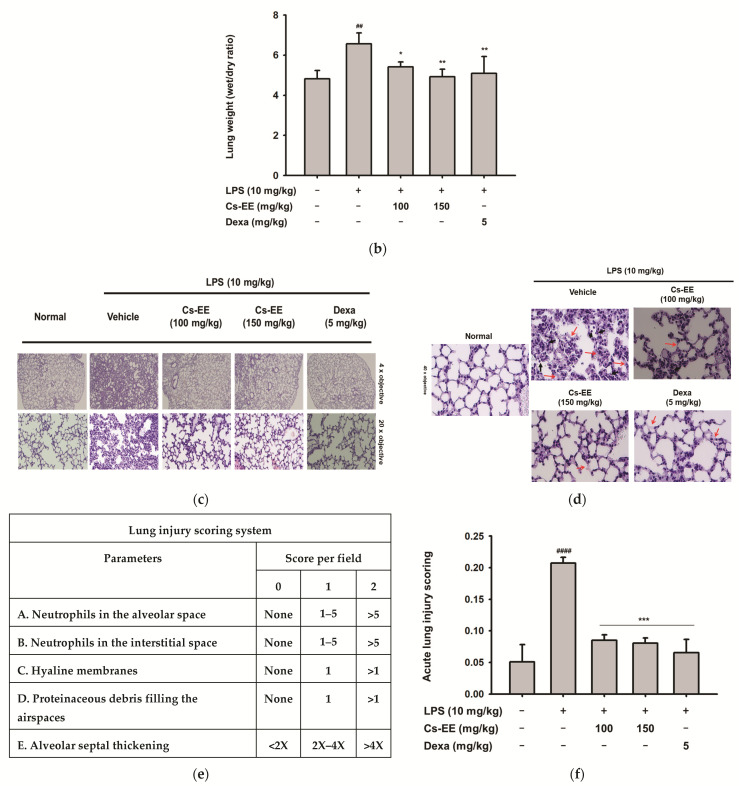

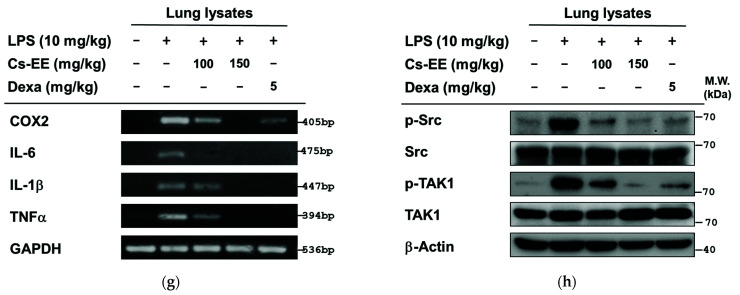
Effects of Cs-EE in the in vivo LPS-induced acute lung injury models. (**a**) Schematic of LPS-induced ALI protocol. C57BL/6 mice were orally treated with 100 μL different solutions based on different groups: normal group (PBS), LPS group or as control group (PBS), Cs-EE groups (100 and 150 mg/kg Cs-EE) and dexa group (5 mg/kg dexa). Acute lung injury was induced by 50 μL intranasal LPS (10 mg/kg) and mice were sacrificed after 12‒16 h. (**b**) The severity of pulmonary edema was evaluated by measuring the lung wet/dry ratio. (**c**–**f**) The representative histopathological examination of lung tissue (H&E staining) was viewed with 100× or 400× magnification (**c**,**d**). The control group showed the thickest of alveolar walls than others; a black arrow indicates neutrophils infiltration, red arrow: hyaline membrane. Histologic ALI scoring system criteria (**e**) were calculated as follows: [(20 × A) + (14 × B) + (7 × C) + (7 × D) + (2 × E)]/(number of fields × 100) (n field = 4). Proteinaceous debris filling the airspaces were considered as none due to the short time dosage. The histology score analyses of acute lung injury were determined using the indicated parameters (**f**). (**g**) The expression of inflammatory genes in lung lysates was analyzed by semiquantitative RT-PCR. (**h**) The phospho- and total forms of Src, TAK1, and β-actin were evaluated by immunoblot analysis. Results (**b**,**f**) are expressed as mean ± SD (n = 4–5). ^##^ *p* < 0.01 and ^####^ *p* < 0.0001 compared to normal group by the student’s *t*-test, and * *p* < 0.05, ** *p* < 0.01 and *** *p* < 0.001 compared to control group (inducer alone) by one-way ANOVA.

Next, we evaluated the anti-inflammatory efficacy of Cs-EE using another inflammatory model, the LPS-induced ALI mouse model, which was created by nasal inhalation, as illustrated in Figure 7a. LPS-induced ALI elevated permeability and lung water level as demonstrated by increased wet-to-dry weight ratios (*p* = 0.002), and Cs-EE treatment together with a standard drug, dexamethasone (dexa), relieved the pulmonary edema (Figure 7b). Furthermore, we assessed the histopathology of lung tissues by HE staining, then quantitatively scored the ALI. As indicated in Figure 7c,d, the normal lung showed thin alveolar walls, few neutrophils, as well as hyaline membranes. In contrast, LPS-induced ALI showed enhanced neutrophil infiltration, marked thickening of alveolar walls, and multiple formation of hyaline membrane. Cs-EE- and dexa-treated groups strongly alleviated the lung injury as measured by a standardized histology score (Figure 7e,f). In addition, in lung samples, the levels of inflammatory genes were also dose-dependently decreased by Cs-EE (Figure 7g). We also examined phosphorylated Src and TAK1 in lung lysates and found that p-Src and TAK1 were strongly inhibited by treatment with Cs-EE, as in the gastritis models (Figure 7h).

## 3. Discussion

Starting from groups of plants that are widely used in traditional medicines, authenticating as well as understanding the mechanisms of therapeutic action could lead to the discovery of new drugs from natural sources with reduced or no side effects. One such group of plants that is implicated in the treatment of different diseases in various parts of the world is that belonging to the genus *Cissus*. The genus *Cissus* consists of about 350 species, of which several species have been described to treat many illnesses including inflammatory disease [15,16,17,18]. The therapeutic use of the genus *Cissus* inspired us to describe the molecular mechanism of one of its species, as the pharmacological mechanisms of *C. subtetragona* have not yet been reported. However, this plant has been used medicinally by folk healers. In the present study, activated macrophages exposed with LPS, a classical stimulus to induce an M1 macrophage phenotype [19], showing higher expression of pro-inflammatory genes such as iNOS, IL-6, and TNF-α, and murine models of acute gastritis and lung injury were utilized to explore the potential anti-inflammatory mechanism of Cs-EE. For the first time, it was found that Cs-EE can have a clinical potential to ameliorate M1 macrophage-mediated inflammatory diseases, since the expression of several M1 macrophage-specific pro-inflammatory genes was strongly reduced by this extract [20]. 

As initially hypothesized, Cs-EE reduced the secretion of NO and PGE_2_ as well as downregulated the expression of inflammatory genes in activated macrophages, suggesting that the inhibition of these inflammatory mediators occurred at the transcriptional level (Figure 1). Importantly, Cs-EE produces these effects without affecting cell viability, which suggests that the inhibitory effects are not due to non-specific toxicity. These suppressed inflammatory responses are the outcome of attenuated Src and TAK1 activation by Cs-EE in the NF-κB and AP-1 pathways (Figure 3). Varied experimental approaches, including luciferase assays, immunoblot analysis, overexpression strategies, and CETSA, identified the targeting of Src and TAK1, which play a crucial role in the activation of NF-κB or AP-1, as the anti-inflammatory mechanism of Cs-EE. The results of the luciferase reporter genes indicated that Cs-EE regulates the mRNA level of cytokines by regulating the transcriptional activity of NF-κB and AP-1. Strengthening these findings, immunoblot analysis of the level of phosphorylation of NF-κB and AP-1 subunits revealed that Cs-EE modulates the transcriptional activation of NF-κB and AP-1. Besides NF-κB and AP-1 subunits, other upstream signaling proteins have been identified that are involved in the NF-κB and AP-1 pathways, including IκBα and PI3K p85 or MAPKs and MAPKKs, respectively. In agreement with another report [21], the IκBα and p-IκBα bands were not regularly detected at 15 min due to the degradation, while rapid recovery of the protein at 30 min after LPS stimulation was observed [22]. Since Cs-EE regulated IκBα at 5 min, we examined whole-cell lysates from LPS-stimulated RAW264.7 cells or primary peritoneal macrophages at earlier time points. The protein levels of p-Src were reduced along with its downstream protein, p85. In line with this, the levels of p-IκBα were also decreased before and at 5 min. Accordingly, Src could be a putative target of Cs-EE in the NF-κB pathway (Figure 4). Numerous studies have demonstrated that Src has a crucial role in activating immune cells and is part of upstream signaling enzymes that mediate transcriptional activation of NF-κB via phosphorylation of PI3K p85 then IKK to degrade IκBα. Indeed, anti-inflammatory remedies derived from compounds or plant extracts that target Src have been shown to diminish inflammatory responses via a blockade of NF-κB pathways [10,11,22]. Furthermore, Cs-EE inhibited activation of two MAPKs, JNK and ERK. Consequently, we checked signaling molecules upstream of these MAPKs, and Cs-EE specifically inhibited MKK4 at the earlier time point. Next, Cs-EE treatment inhibited TAK1 activation. Consequently, TAK1 could be a putative target in the AP-1 pathway (Figure 5). Previous studies reported the essential role and mechanism of TAK1 in activating AP-1 pathways. Upon stimulation, TAK1 could activate the MAPKKs (MKK3/6 and MKK4/7) to transduce the signal to MAPKs (p38, JNK, and ERK) [23], although this protein is also involved in the activation of the NF-κB pathway. In addition, anti-inflammatory remedies derived from compounds or plant extracts that target TAK1 have been reported to inhibit inflammatory responses via a blockade of AP-1 pathways [24,25,26]. Using overexpression strategies and CETSA assays, we confirmed that Src and TAK1 were targeted by Cs-EE as well as gained insight into the interaction of Cs-EE with its target proteins. Following previous studies, Src- or TAK1-overexpressing HEK293 cells expressed autophosphorylation events of Src and TAK1, respectively [24,26]. Treatment with Cs-EE reduced protein expression of phosphorylated Src and TAK1 as well as led to the thermo-stabilization of Src and TAK1. 

In line with the in vitro studies, in vivo experiment conditions mimicked the anti-inflammatory activity of Cs-EE via attenuation of Src- and TAK1-mediated pathways, thereby inhibiting expression of inflammatory genes as well as alleviating the inflammatory manifestations. Most standard anti-inflammatory drugs, including corticosteroids and nonsteroidal anti-inflammatory drugs (NSAID) such as dexamethasone, aspirin, indomethacin, or ibuprofen, may occasionally cause gastrointestinal inflammation [27]. For this reason, we explored the anti-inflammatory effect of Cs-EE in the HCl/EtOH-induced acute gastritis model to determine whether it could provide a safer and more efficient anti-inflammatory remedy (Figure 6). Injection of HCl/EtOH induces mucosal injury of the stomach which leads to DAMPs recognition and subsequent stimulation of inflammatory cytokines such as IL-1β, IL-6, and TNF-α [1,28]. As expected, Cs-EE treatment decreased inflammatory responses, and exhibited the protective effects on gastric mucosa as that of a standard gastroprotection drug to prevent gastric ulcers, ranitidine. Thus, Cs-EE treatment exhibited anti-inflammatory properties as well as gastric protection. Moreover, to explore the efficacy of Cs-EE in another inflammatory disease, the LPS-induced ALI models were utilized. To define ALI in animal models as in humans, various alternative approaches were employed, including the use of histopathological criteria such as inflammatory infiltrates, thickened alveolar septae, and deposition of hyaline membranes [29]. In agreement with previous studies, LPS-induced ALI models showed injured lungs, increased lung wet/dry weight ratios, and enhanced expression of inflammatory genes as well as Src and TAK1 activation. In contrast, Cs-EE and dexa treatment inhibited the inflammatory responses (Figure 7). These findings support that orally administered Cs-EE was able to alleviate the disease manifestations and may be clinically beneficial for treating various inflammatory problems. 

A variety of flavonoid molecules show anti-inflammatory activity in vitro and in vivo via several mechanisms, including inhibition of the expression of various inflammation-related proteins/enzymes by suppressing activation of transcription factors such as NF-κB and AP-1 [30]. Of the many flavonoid classes that exist, Cs-EE contains the bioactivity of the flavonols kaempferol and isorhamnetin, isoflavones daidzin and genistin, flavanone naringenin, flavone apigenin, as well as biflavone 5′-methoxybilobetin that possess remarkable anticancer, antimicrobial, immunomodulatory, and anti-inflammatory effects. Some studies have demonstrated that kaempferol, isorhamnetin, genistin, daidzein, naringenin, and apigenin regulate the production of several cytokines as well as inflammatory mediators, including NO and PGE_2_, attenuate expression of inflammatory genes, and inhibit NF-κB or AP-1 activation in activated macrophages, LPS-stimulated microglial cells, and various other cell types [30,31,32,33,34]. Consistently, we found that kaempferol, genistin, and apigenin showed inhibitory effects on NO production in LPS-stimulated RAW264.7 cells (Figure 2). Consequently, we considered that these components of Cs-EE could be responsible for its anti-inflammatory effect.

## 4. Materials and Methods

### 4.1. Materials

HEK293 cells (ATCC number CRL-1573) and RAW264.7 cells (ATCC number TIB-71) were acquired from the American Type Culture Collection (ATCC) (Rockville, MD, USA). Dimethyl sulfoxide (DMSO), carboxymethylcellulose (CMC), 3-(4,5-dimethylthiazol,2-yl)-2,5-diphenyltetrazolium bromide (MTT), polyethylenimine (PEI), sodium dodecyl sulfate (SDS), ranitidine, dexamethasone (dexa), L-NAME, kaempferol, genistin, apigenin, lipopolysaccharide (LPS, E. coli 0111:B4), pam3csk4, and poly (I:C) were obtained from Sigma-Aldrich Co. (St. Louis, MO, USA). RPMI 1640, DMEM, trypsin, PBS, and penicillin-streptomycin were received from HyClone (Logan, UT, USA). Fetal bovine serum (FBS) was acquired from Biotechnics Research, Inc. (Irvine, CA, USA). TRIzol reagent was purchased from MRCgene (Cincinnati, OH, USA). Primers used for semiquantitative RT-PCR and qPCR were purchased from Macrogen Inc. (Seoul, Korea). Phospho-specific or total-protein antibodies against p65, p50, IκBα, PI3K/p85, Src, c-Fos, c-Jun, JNK, ERK, p38, MKK4, MKK7, MEK1/2, TAK1, IRAK1, IRAK4, hemagglutinin (HA), and β-actin were acquired from Cell Signaling Technology (Beverly, MA, USA) and Santa Cruz Biotechnology (Santa Cruz, CA, USA). Constructs for signaling proteins HA-Src and HA-TAK1 were used as previously reported [24,26]. 

### 4.2. Plant Collection, Specimen Information, and Cs-EE Preparation

Cs-EE (Code number: NIBR 1063) was acquired from National Institute of Biological Resources (Incheon, Korea). The fresh aerial parts of C. subtetragona Planch. were collected in the Volikhamsai region, Laos, on September 27, 2018. The collections were done by the project team (Prof. Woo Shin Lee and Dr. Ji-Hwa Lee). Using an ultrasonic extractor (Ultrasonic Cleaner UC-10, UC-20, 400 W), 70% ethanol was poured to cover the dried branch with leaves of C. subtetragona (960 g) for 3 h at 50 °C (3 times) to yield 10.83 of extract. After removal of the solvent under reduced pressure *in vacuo*, the extract was freeze-dried for 48 h at -80 °C and then stored in a freezer at -20 °C until use. During the in vitro studies, the Cs-EE stock solution was made by dissolving the powdered Cs-EE stock with DMSO at a concentration of 100 mg/mL. When each experiment was performed, the stock solution was diluted to the desired final concentration of 25–150 μg/mL using the suitable culture medium. For the gastritis and acute lung injury mouse model experiments, the stock of Cs-EE was prepared in 0.5% CMC and PBS, respectively, at doses of 100 mg/kg and 150 mg/kg. The doses and experimental scheme of Cs-EE in this study were determined, according to previously published papers showing similar in vitro and in vivo activities [10,11,22,24,35].

### 4.3. Cell Culture and Treatment 

The human embryonic kidney cell line (HEK293) and the murine macrophage cell line (RAW264.7) were cultured as previously described [25]. Cs-EE groups were pre-treated with Cs-EE (25–150 μg/mL), while the inducer [poly (I:C), pam3csk4, or LPS] and normal groups were pre-treated with diluted DMSO in culture medium for 30 min. The final level of DMSO in cellular experimental conditions was <0.2%.

### 4.4. Animals

ICR mice (5 weeks old, 18–20 g, male) and C57BL/6 mice (5 weeks old, 17–19 g, male) were acquired from Daehan Biolink (Chungcheonbuk, Korea). The mice (5 per group) accessed water and a pelleted diet (Samyang, Daejeon, Korea) ad libitum in separate cages under a 12-h light/dark cycle. In acute lung injury model, C57BL/6 mouse strain was used since this mouse strain is more sensitive to pulmonary edema and was well demonstrated in the pathogenesis of acute lung injury, according to previous papers [36,37,38]. In contrast, ICR mice have been utilized to conduct gastric ulcer experiments [10,11,22,24]. The in vivo experiments were performed in agreement with the guidelines of the Institutional Animal Care and Use Committee Sungkyunkwan University (Suwon, Korea; approval ID: SKKUIACUC2021-03-07-2 and SKKUIACUC2021-03-56-1).

### 4.5. Preparation of Peritoneal Macrophages

Primary macrophages were obtained from peritoneal exudates of ICR mice by injection of 4% sterile thioglycollate broth (Difco Laboratories, Detroit, MI, USA), as previously described [24]. The cells were then plated (3.75 × 10^6^ cells/mL) into 96-wells and 3-mm^2^ plates for NO assays as well MTT assays and immunoblotting analysis, respectively.

### 4.6. Determination of NO and PGE_2_ Production

RAW264.7 cells (1 × 10^6^ cells/mL) or peritoneal macrophages (8 × 10^6^ cells/mL) were seeded in 96-well-plate and then pre-treated with different concentrations of testing extract or compounds (Cs-EE, L-NAME, kaempferol, genistin, or apigenin) for 30 min. Inflammatory inducers [LPS (1 μg/mL), poly (I:C) (200 μg/mL), or pam3csk4 (10 μg/mL)] were then treated for additional 24 h [39]. The inhibitory effects of Cs-EE, L-NAME, kaempferol, genistin, and apigenin on NO production were detected using Griess reagents, as previously described [40]. Meanwhile, the inhibitory effects on PGE_2_ production were analyzed using an EIA kit according to the manufacturer’s instructions (RnD systems, lot P291245, catalog no. KGE0048).

### 4.7. Cell Viability Assay

The cytotoxic effects of Cs-EE were analyzed by a conventional MTT assay as previously described [41,42]. Pre-incubated RAW264.7 cells, HEK293 cells, and peritoneal macrophages for overnight (16 h) were further treated with 50 μL of Cs-EE (final concentrations: 50, 100, and 150 μg/mL) or DMSO (for normal or control group) for additional 24 h in 96-well plates.

### 4.8. Liquid Chromatography-Tandem Mass Spectrometry (LC-MS/MS)

LC-MS/MS analyses were performed with a Xevo G2-XS LC/QTOF-MS (Waters, Milford, MA, USA) and data collection and analysis used Waters LC/QTOF-MS MassLynx Software ver. 4.2 and Waters UNIFI portal software, respectively (Waters, Milford, MA, USA). The mobile phase is 0.1% formic acid in water (A) and acetonitrile (B), while the UPLC column is the reverse-phase BEH C18, 2.1 × 100 mm, 1.7 μm. The setting of the gradient program was maintained for 2.9 min after conducting 20–95% B at 0–14 min, 95–100% B at 14–15 min, and 100–20% B at 15–15.1 min. The setting of column temperature was conducted at the flow rate of 0.3 mL/min (45 °C) with 2 μL of the injection volume. The mass spectrometry was established with status: gas temperature at 150 °C, 50 L/h flow rate of N2 gas, 700 L/h flow rate of nebulizer gas, and 1.2 kV capillary voltage. The mass spectrum range from 40–1200 *m/z* was obtained using the positive ESI mode.

### 4.9. mRNA Analysis by Quantitative Real-Time (q) Polymerase Chain Reaction (PCR) and Semiquantitative Reverse Transcriptase (RT)-PCR

Total RNA was extracted from macrophage-like RAW264.7 cells (1 × 10^6^ cells/mL) that were pre-treated with Cs-EE (0, 100, and 150 μg/mL) for 30 min followed by incubation with LPS (1 μg/mL) for 6 h. Stomach and lung tissues of mice were also collected, immediately frozen, and stored at −70 °C. Total RNA was isolated using TRIzol as stated by the manufacturer’s instructions, and then 1 μg of total RNA was immediately prepared for cDNA synthesis using a cDNA synthesis kit (Thermo Fisher Scientific, Waltham, MA USA) as stated by the manufacturer’s instructions. The qPCR and semiquantitative RT-PCR were conducted as previously reported [43,44]. The primer sequences utilized in this study are listed in Table 1.

### 4.10. Plasmid Transfection and Luciferase Reporter Gene Assay

HEK293 cells (2 × 10^5^ cells/mL) were pre-incubated overnight before transfection of plasmids encoding NF-κB and AP-1 reporter genes under co-transfection conditions with MyD88 and TRIF, respectively, by polyethylenimine (PEI) methods [45]. After 24 h stabilization, the transfected cells were treated with Cs-EE for the next 24 h. Luciferase activity was determined using a Luciferase Assay System (Promega, Madison, WI, USA). To evaluate the autophosphorylation of Src and TAK1, HEK293 cells (1 × 10^6^ cells/mL) were transfected with Src- and TAK1-encoding genes, respectively, for 24 h. The cells were additionally treated with Cs-EE for the next 12 h. The level of phosphorylated Src and TAK1, HA (tag protein), and β-actin were visualized from whole-cell lysates of Src- and TAK1-transfected cells by immunoblotting analysis.

### 4.11. Cell Lysate Extraction and Immunoblotting Analysis

Cells or stomach and lung tissues of mice were lysed in lysis buffer as previously described [22]. Around 20–40 μg of protein were separated on SDS/polyacrylamide electrophoresis and then transferred onto an immune-blot PVDF membrane. The membranes were incubated with primary antibodies followed by conjugated secondary antibodies (anti-mouse or anti-rabbit IgG-horseradish peroxidases) according to the manufacturer’s instructions after blocking with 3% BSA in Tris-buffered saline with tween 20. Proteins were detected using WSE-7120 EzWestLumi plus (ATTO, Tokyo, Japan) with ChemiDoc ATTO WSE-6300 LuminoGraph III (ATTO, Japan).

### 4.12. Cellular Thermal Shift Assay (CETSA)

Pre-incubated HEK293 cells (2 × 10^6^ cells/mL) were made to overexpress either Src or TAK1 using the PEI transfection method. After 24 h, the cells were treated with Cs-EE (150 μg/mL) or DMSO for 12 h. Then, a CETSA assay was performed as previously described [10] and cell lysates were subjected to immunoblotting.

### 4.13. HCl/EtOH-Induced Acute Gastritis

Using ICR mice (5 mice/group), 60% EtOH in 150 mM HCl was orally delivered to induce acute gastritis, according to the previously published method [46,47]. To begin starvation, the bedding and food were removed starting from 24 h before compound treatment. Fasted ICR mice were orally administrated with 100 μL different solutions based on different groups: Normal group (0.5% CMC), control group (0.5% CMC + HCl/EtOH), compound treatment groups (100 and 150 mg/kg Cs-EE + HCl/EtOH), and ranitidine group (40 mg/kg ranitidine + HCl/EtOH). Eight hours later, a second oral administration was performed, followed by a final oral administration after 16 h. After 6 h, 200 μL freshly prepared 60% EtOH/150 mM HCl was orally administrated to each group, except normal group (orally administrated with 200 μL 0.5% CMC). One hour after, mice were sacrificed by CO_2_ exposure. Then, the stomach was isolated, gently washed with PBS, opened along the greater curvature, and photographed with a white background. Subsequently, the redness of gastric mucosal lesions was observed, the inflamed area (mm^2^) with mucosal erosive lesions was measured using pixel counter with custom ImageJ-based software, and the tissues were stored at -−70°C until used for RNA extraction and protein analysis.

### 4.14. LPS-Induced Acute Lung Injury

Using C57BL/6 mice (5 mice/group), acute lung injury was induced by the nasal inhalation of LPS (10 mg/kg) as previously reported [29]. The mice were orally treated with PBS, Cs-EE or dexa 3 times for one day based on different groups. One hour before the final administration, LPS was intranasally administered, except normal group was intranasally administered with PBS. After sacrifice, the lungs were excised and rinsed with PBS. The left side was directly evaluated for pulmonary edema by using the lung wet/dry ratio (n = 5). Meanwhile, the upper right side was immersed in 10% formalin for the following histopathological examination (n = 5) with hematoxylin and eosin (HE) staining using a previously reported method [48]. The evaluations were performed as in a previous study [29] and the lower right side of the lung tissues (n = 5) were stored at −70 °C until used for RNA extraction as well as protein analysis.

### 4.15. Statistical Analysis

All scores in this study represent the mean ± SD of three samples (enzyme assay), five samples (in vitro experiments) and five mice per group (in vivo experiments). Similar experimental in vitro and in vivo data were obtained from an additional independent experiments performed under same conditions. Statistical analyses was performed using SigmaPlot (Systat Software, San Jose, CA, USA), in which a comparison of statistical differences of all measured data (treated groups vs. control group) was subjected to one-way ANOVA followed by Holm–Sidak, meanwhile, a comparison of the two groups (control group vs. normal group) was analyzed using the student’s t-test. A *p*-value of *p* < 0.05 was considered to be a statistically significant difference. 

## 5. Conclusions

**Figure 8:**
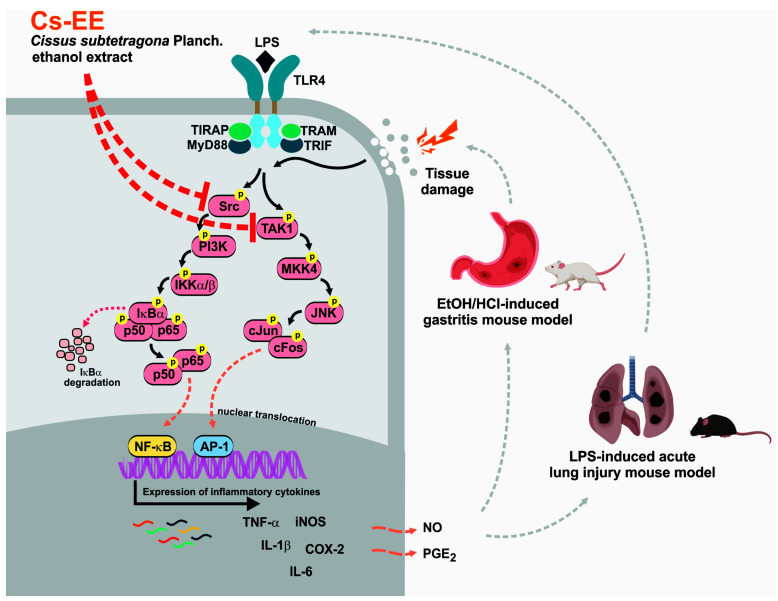
Schematic: anti-inflammatory mechanisms of Cs-EE in the inhibition of NF-κB and AP-1 signaling pathways via attenuation of Src and TAK1 activation.

The present study, using in vitro and in vivo systems, validated the anti-inflammatory potential of Cs-EE and identified that Cs-EE targets Src and TAK1, as summarized in Figure 8, thereby diminish the activity of NF-κB and AP-1. The anti-inflammatory activity of *C. subtetragona* Planch. is attributed to the presence of bioactive molecules that have demonstrated this function in various other studies. In the future, the development of anti-inflammatory therapies from natural plants, especially those derived from the extract *C. subtetragona* Planch., could be promising agents for preventing or relieving inflammatory manifestations such as gastritis and acute lung injury. Further investigations are needed to identify and isolate the bioactive molecules present in *C. subtetragona* Planch. as well as to validate *C. subtetragona* Planch. in clinical trials.

## Figures and Tables

**Table 1:** Primer sequences used for PCR.

PCR Type	Genes Name	Sequence (5′-3′)	
Semiquantitative RT-PCR	*GAPDH*	Forward	GAAGGTCGGTGTGAACGGAT
		Reverse	AGTGATGGCATGGACTGTGG
	*iNOS*	Forward	CAAGAGAACGGAGAACGGAGA
		Reverse	GATGGACCCCAAGCAAGACT
	*COX-2*	Forward	TGAGTACCGCAAACGCTTCT
		Reverse	TGGGAGGCACTTGCATTGAT
	*TNF-α*	Forward	TTGACCTCAGCGCTGAGTTG
		Reverse	CCTGTAGCCCACGTCGTAGC
	*IL-6*	Forward	GCCTTCTTGGGACTGATGCT
		Reverse	TGGAAATTGGGGTAGGAAGGAC
	*IL-1β*	Forward	CAGGATGAGGACATGAGCACC
		Reverse	CTCTGCAGACTCAAACTCCAC
qPCR	*GAPDH*	Forward	GAAGGTCGGTGTGAACGGAT
		Reverse	AGTGATGGCATGGACTGTGG
	*iNOS*	Forward	CAAGAGAACGGAGAACGGAGA
		Reverse	GATGGACCCCAAGCAAGACT
	*COX-2*	Forward	TGAGTACCGCAAACGCTTCT
		Reverse	TGGGAGGCACTTGCATTGAT
	*TNF-α*	Forward	TTGACCTCAGCGCTGAGTTG
		Reverse	CCTGTAGCCCACGTCGTAGC
	*IL-6*	Forward	GCCTTCTTGGGACTGATGCT
		Reverse	TGGAAATTGGGGTAGGAAGGAC
	*IL-1β*	Forward	CAGGATGAGGACATGAGCACC
		Reverse	CTCTGCAGACTCAAACTCCAC

## Data Availability

The data used to support the findings of this study are available from the corresponding author upon request.

## Supplementary Materials

The following are available online, Supplementary Table S1: LC-MS/MS of Cs-EE.

## Sample Availability

Sample of the compound Cs-EE is available from the authors.

## Abbreviations

*TAK1*	Transforming growth factor-β-activated kinase 1
*NF-* *κB*	Nuclear factor-κB
*AP-1*	Activator protein-1
*MAPKs*	Mitogen-activated protein kinases
*JNK*	c-Jun N-terminal kinase
*PI3K*	Phosphoinositide 3-kinase
*PGE_2_*	Prostaglandin E_2_
*COX-2*	Cyclooxygenase-2
*IL-1* *β*	Interleukin-1β
*IL-6*	Interleukin 6
*TNF-α*	Tumor necrosis factor-alpha
*MKK*	Mitogen-activated protein kinase
*iNOS*	Inducible nitric oxide synthase
