# Supplementary material for: Cissus subtetragona Planch. Ameliorates Inflammatory Responses in LPS-induced Macrophages, HCl/EtOH-induced Gastritis, and LPS-induced Lung Injury via Attenuation of Src and TAK1
Source: Molecules. 2021 Oct 8;26(19):6073. doi: 10.3390/molecules26196073 (PMC8512965; doi:10.3390/molecules26196073)
Supplement: Supplementary file 1 [file molecules-26-06073-s001.zip › molecules-1378091-supplementary.pdf]

Supplementary Table S1 (LC-MS/MS of Cs-EE)

<아모레퍼시픽 Flavonoid Q-Tof 결과>

***Cissus subtetragona* Planch. ethanol extract**

No.	Component name	Formula	Observed RT (min)	Mass error (ppm)	Total Fragments Found	Isotope Match Mz RMS PPM	Isotope Match Intensity RMS Percent	Detector counts	Response	Adducts	Neutral mass (Da)	Observed neutral mass (Da)
70	5,6,3',4'-Tetrahydroxy-3,7-demethoxy flavone	C17H14O8	0.82	0.1	16	14.96	17.00	31214	31214	+Li	346.06887	346.0689
51	3',5',β-Trihydroxy-3,4,4',α-tetramethoxychalcone	C19H20O8	0.85	-2.0	47	11.39	559.48	7136	7136	+K	376.11582	376.1150
3	(2R,3R)-3,5,7,2',6'-Pentahydroxy flavanone	C15H12O7	1.06	-2.5	19	51.97	32.93	53712	53712	+Li	304.05830	304.0575
18	1,5-Dihydrxy-2,3,4,7-tetramethoxyxanthone	C17H16O8	1.06	-1.8	18	9.09	22.85	39718	24946	+Li	348.08452	348.0839
276	Limocitrin-3-O-β-D-glucopyranoside	C23H24O13	1.06	-2.7	97	5.28	118.45	62002	29695	+H	508.12169	508.1203
20	2,5-Dimethyl-7-hydroxychromone	C11H10O3	1.07	-3.1	20	23.95	175.67	8809	8809	+Li	190.06299	190.0624
202	Isorhamnetin	C16H12O7	1.08	-1.0	24	43.67	47.81	40837	25055	+Li	316.05830	316.0580
327	Odoratin-7-O-β-D-glucoside	C22H24O9	1.08	1.1	127	7.04	43.66	109431	60852	+H	432.14203	432.1425
195	Irilone	C16H10O6	1.32	1.7	2	2.74	303.90	12419	12419	+Li	298.04774	298.0482
75	5,7,2'-Trihydroxy-6-methyl-8-methoxy-3(R)-(4'-methoxybenzyl) chroman-4-one	C19H20O7	1.40	1.2	34	2.72	542.08	13500	13500	+K	360.12090	360.1214
336	Phellochinin A	C26H30O11	1.50	-0.1	65	5.54	189.03	129495	17695	+H	518.17881	518.1787
80	5,7,8,2'-Tetrahydroxy-flavone-7-O-β-D-glucoside	C21H20O11	1.60	-3.9	30	4.98	2469.26	19666	11746	+Na, +H	448.10056	448.0987
160	Cyclomorusin	C25H22O6	1.64	-3.5	14	3.55	439.90	129735	107187	+Li	418.14164	418.1401
32	2''-Acetylastragalin	C23H22O12	1.65	3.7	42	4.67	3284.92	7067	7067	Sn	490.11113	490.1134
69	4',5,7-Trihydroxy-3,6-dimethoxyflavone-7-O-β-D-glucopyranoside	C23H24O12	1.67	0.6	44	2.89	112.29	37100	23478	+Li	492.12678	492.1271
71	5,6,4'-Trihydroxy flavone-7-O-β-D-galactonic acid	C22H22O11	1.68	2.3	26	18.73	74.52	104434	25980	+Na	462.11621	462.1173
295	Methyl ophiopogonanone A	C19H18O6	1.69	-3.0	14	4.66	109.44	31431	26305	+Li	342.11034	342.1093
342	prim-O-Glucosylcimifugin	C22H28O11	1.71	1.4	32	2.15	65.86	135773	22086	+Na	468.16316	468.1639
376	Sanggenon G	C40H38O11	1.72	3.8	43	3.43	195.23	45554	19630	+H	694.24141	694.2441
223	Isosilybin	C26H24O10	1.74	2.0	26	2.03	234.62	155336	51784	+Li	496.13695	496.1379
74	5,7,2',3',4'-Pentamethoxyflavone	C20H20O7	1.77	1.4	14	51.44	11.87	241822	126970	+Li, +Na	372.12090	372.1214
81	5,7-Dihydroxy-6,8-dimethyl-3-(2'-hydroxy-3',4'-methylenedioxy-benzyl)chromone	C19H16O7	1.77	-0.7	12	1.47	114.97	32515	26698	+Li	356.08960	356.0893
166	Diosmetin 3'-O-α-D-glucoside	C22H22O11	1.77	0.9	29	1.56	387.41	200612	86793	+Li, +Na	462.11621	462.1167
57	3,8-Dihydroxy-4,10-dimethoxy-7-oxo-[2] benzopyrano[4,3-b] [1]benzopyran-7-(5H)-one	C18H14O7	1.82	-4.3	13	27.88	25.66	53857	44154	+NH4	342.07395	342.0724
197	Isoaloeresin D	C29H32O11	1.83	1.4	98	18.64	43.82	1730981	1240075	+Li	556.19446	556.1952
232	Kaempferol-3-Glucoside-2''-p-coumaroyl	C30H26O13	1.86	1.5	56	1.74	1998.36	634940	429250	+Li, +Na	594.13734	594.1382
388	Sophorabioside	C27H30O14	1.86	2.9	94	2.77	4.00	1398768	447856	+H, +Na, +K	578.16356	578.1652
282	Lupinifolin	C25H26O5	10.73	-3.8	7	58.49	321.80	60866	15891	+Li	406.17802	406.1765
260	Kushenol X	C25H28O7	11.79	-4.4	8	7.12	28.59	202918	151496	+H	440.18350	440.1816
201	Isoanhyoicaritin	C22H24O6	12.49	-1.7	27	33.55	31.87	45674	36914	+H	384.15729	384.1566
39	2-Methoxykurarinone	C27H32O6	14.00	3.4	22	4.26	710.14	10066	10066	+Na	452.21989	452.2215
320	Neocomplanoside	C24H24O12	14.31	2.9	97	3.81	1050.26	16779	16779	+NH4	504.12678	504.1283
281	Liquiritin apioside	C26H30O13	14.32	2.8	173	4.28	137.20	105383	40223	+Na, +K	550.16864	550.1702
333	Paeonin	C28H33ClO16	14.46	1.2	36	3.19	2313.63	37126	19606	+Li, +Na	660.14571	660.1465
88	5'-Methoxy-bilobetin	C32H22O11	15.37	0.0	17	0.37	853.00	16544	9949	+K	582.11621	582.1162
313	Mururin A	C24H16O9	2.06	-0.1	7	1.23	125.80	45113	28213	+Li	448.07943	448.0794
181	Genistin	C21H20O10	2.08	2.4	36	2.71	14.96	147001	78403	+H, +Na	432.10565	432.1067
234	Kaempferol-3-glucuronide	C21H20O12	2.09	-0.4	22	2.37	426.91	9261	9261	+Na	464.09548	464.0953
105	Acacetin-7-O-(6''-O-acetyl)-β-D-glucopyranoside	C24H24O11	2.22	0.0	26	2.45	822.60	25666	13729	+Na, +H	488.13186	488.1319
338	Pinnatifine I	C23H20O10	2.23	-1.4	20	2.07	52.40	31648	26063	+H	456.10565	456.1050
68	4,7,2'-Trihydroxy-4'-methoxyisoflavanol	C16H16O5	2.33	0.7	10	4.09	16.81	45425	25254	+H, +Na	288.09977	288.1000
138	Baohuoside Ⅰ	C27H30O10	2.37	0.8	27	8.35	56.64	88623	27467	+Na	514.18390	514.1843
372	Sanggenon A	C25H24O7	2.37	0.0	16	1.26	253.32	88360	58596	+Li, +Na	436.15220	436.1522
252	Kushenol O	C27H30O13	2.55	0.8	48	1.82	37.98	88399	38006	+H	562.16864	562.1691
129	Apigenin-7-O-rhamnoside	C21H20O9	2.66	-0.4	12	1.57	107.88	29366	24049	+Li	416.11073	416.1106
114	Aloeresin G	C29H30O10	2.67	0.7	43	13.86	14.00	101861	49578	+Li, +Na	538.18390	538.1843
12	(3R,4S)-3,4-Dihydroxy-3-(3',4'-dimethoxybenzyl)-7-methoxy-chroman	C19H22O6	2.85	1.7	12	2.20	26.24	277929	28343	+Na	346.14164	346.1423
31	2',6'-Dihydroxy-4'-methoxy-dihydrochalcone	C16H16O4	2.87	0.2	6	6.60	46.31	95155	44369	+Na, +H, +K	272.10486	272.1049
134	Artemitin	C20H20O8	3.05	-0.6	2	1.12	34.90	34047	27913	+Na	388.11582	388.1156
227	Kaempferol	C15H10O6	3.11	1.6	4	20.94	1.17	86182	74271	+H	286.04774	286.0482
334	Patuletin-7-O-[6''-(2-methylbutyryl)]-glucoside	C26H28O14	3.11	2.0	31	2.34	6.43	358359	201491	+H, +Na	564.14791	564.1490
402	Tribuloside	C30H26O13	3.36	0.9	33	1.36	426.42	126943	90377	+Li	594.13734	594.1379
279	Liquiritigenin-4'-O-β-D-glucopyranoside	C21H22O9	3.39	0.1	28	0.26	49.67	50589	36136	+Na	418.12638	418.1264
314	Naringenin	C15H12O5	3.39	-0.8	7	20.58	10.70	116480	50676	+H, +Na, +K	272.06847	272.0683
128	Apigenin-7-O-acetyl-β-D-glucoside	C23H22O11	3.62	0.5	6	2.47	468.36	8748	8748	+H	474.11621	474.1165
149	Chebuloside Ⅱ	C36H58O11	3.64	-1.8	18	2.27	27.63	49178	29211	+Na	666.39791	666.3966
379	Sappanol	C16H16O6	3.65	-0.9	9	2.50	214.65	7548	7548	+K	304.09469	304.0944
170	DO 21	C32H26O9	3.69	3.6	21	4.48	1117.79	10718	10718	+H	554.15768	554.1597
275	Licurazide	C26H30O13	3.69	-0.1	60	3.77	6757.45	29837	13721	Sn	550.16864	550.1686
304	Mirificin	C26H28O13	3.69	1.6	56	1.38	3.81	4730044	2229424	+H, +Na, +Li	548.15299	548.1539
33	2'-Hydroxy-4,4',6'-trimethoxydihydrochalcone	C18H20O5	3.70	0.8	20	1.46	399.57	136786	102181	+Li, +Na	316.13107	316.1313
132	Apigenol	C15H10O5	3.70	1.2	12	1.12	1.66	1117357	934148	+H	270.05282	270.0531
161	Daidzin	C21H20O9	3.75	0.1	11	3.00	3.33	186435	146025	+H	416.11073	416.1108
98	6-Methoxy-2-(2-phenylethyl)chromone	C18H16O3	3.79	-0.2	6	1.69	171.92	53901	44313	+Li	280.10994	280.1099
294	Methyl kushenol C	C26H28O7	3.92	4.6	10	4.44	147.05	49884	32928	+Li, +Na	452.18350	452.1856
406	Viscumneoside Ⅱ	C25H26O13	4.11	4.2	10	4.89	877.15	12901	12901	+Na	534.13734	534.1397
303	Methyl ophiopogonanone B	C19H20O5	5.37	-3.8	4	6.85	46.16	340179	213767	+H	328.13107	328.1298
76	5,7,3',4'-Tetramethoxyflavone	C19H18O6	6.65	2.7	3	2.59	21.57	40223	19635	+Na, +H	342.11034	342.1113
176	Flavonol	C15H10O3	6.77	3.3	1	4.58	399.78	127114	114302	+Li	238.06299	238.0638
22	2',6'-Dihydroxy-4,4'-dimethoxydihydrochalcone	C17H18O5	6.99	0.4	9	1.48	138.39	52981	34198	+Li	302.11542	302.1156
83	5,7-Dihydroxy-6-methoxyl-8-methyl-3-(4'-methoxybenzyl) chroman-4-one	C19H20O6	7.11	-0.4	10	2.24	385.12	19455	11900	+Na, +H	344.12599	344.1259
251	Kushenol M	C30H36O7	8.30	3.4	10	45.66	56.32	136068	25202	+Li	508.24610	508.2478
46	3-(4'-Hydroxy-benzyl)-5,7-dihydroxy-6,8-dimethyl-chroman-4-one	C18H18O5	8.46	-0.8	7	1.10	27.24	50814	42087	+Na	314.11542	314.1152
140	Bavachromene	C20H18O4	9.81	2.9	5	48.71	16.09	62605	50323	+Li	322.12051	322.1215
